# Clinical and neurophysiological effects of central thalamic deep brain stimulation in the minimally conscious state after severe brain injury

**DOI:** 10.1038/s41598-022-16470-2

**Published:** 2022-07-28

**Authors:** Hisse Arnts, Prejaas Tewarie, Willemijn S. van Erp, Berno U. Overbeek, Cornelis J. Stam, Jan C. M. Lavrijsen, Jan Booij, William P. Vandertop, Rick Schuurman, Arjan Hillebrand, Pepijn van den Munckhof

**Affiliations:** 1 Department of Neurosurgery, Amsterdam Neurosciences, Systems & Network Neurosciences, Amsterdam UMC (Location AMC), University of Amsterdam, Meibergdreef 9, 1105 AZ Amsterdam, The Netherlands; 2 Department of Clinical Neurophysiology and MEG Center, Amsterdam Neuroscience, Amsterdam UMC, Vrije Universiteit Amsterdam, Amsterdam, The Netherlands; 3 Sir Peter Mansfield Imaging Centre, School of Physics and Astronomy, University of Nottingham, Nottingham, UK; 4 Department of Primary and Community Care, Center for Family Medicine, Geriatric Care and Public Health, Radboud University Medical Center, Nijmegen, The Netherlands; 5 Accolade Zorg, Bosch en Duin, The Netherlands; 6 Libra Rehabilitation & Audiology, Tilburg, The Netherlands; 7 Department of Radiology and Nuclear Medicine, Amsterdam UMC, University of Amsterdam, Amsterdam, The Netherlands

**Keywords:** Disorders of consciousness, White matter injury

## Abstract

Deep brain stimulation (DBS) of the central thalamus is an experimental treatment for restoration of impaired consciousness in patients with severe acquired brain injury. Previous results of experimental DBS are heterogeneous, but significant improvements in consciousness have been reported. However, the mechanism of action of DBS remains unknown. We used magnetoencephalography to study the direct effects of DBS of the central thalamus on oscillatory activity and functional connectivity throughout the brain in a patient with a prolonged minimally conscious state. Different DBS settings were used to improve consciousness, including two different stimulation frequencies (50 Hz and 130 Hz) with different effective volumes of tissue activation within the central thalamus. While both types of DBS resulted in a direct increase in arousal, we found that DBS with a lower frequency (50 Hz) and larger volume of tissue activation was associated with a stronger increase in functional connectivity and neural variability throughout the brain. Moreover, this form of DBS was associated with improvements in visual pursuit, a reduction in spasticity, and improvement of swallowing, eight years after loss of consciousness. However, after DBS, all neurophysiological markers remained significantly lower than in healthy controls and objective increases in consciousness remained limited. Our findings provide new insights on the mechanistic understanding of neuromodulatory effects of DBS of the central thalamus in humans and suggest that DBS can re-activate dormant functional brain networks, but that the severely injured stimulated brain still lacks the ability to serve cognitive demands.

## Introduction

Severe acquired brain injury can result in a prolonged disorder of consciousness (PDOC), such as the minimally conscious state (MCS), one of the most dramatic chronic conditions in medicine. MCS is characterized by partial preservation of consciousness, expressed in patients by inconsistent non-reflexive reactions, such as visual pursuit, object manipulation, recognizable yes–no responses, and/or command following^1–3^. MCS is subcategorized, based on the complexity of the observed behaviors, in MCS- with low-level behavioral responses (visual pursuit, localization of noxious stimulation) and MCS + with high-level language-dependent responses (command following, intelligible verbalizations, intentional communication)^4,5^. Although patients show discernable purposeful behavior and exhibit signs of minimal self-awareness and awareness of their environment, they are incapable of adequate and consistent responses to the outside world. Medical care for these patients is mainly supportive, as treatment options remain scarce^6^.

Since the early 1960’s there have been several attempts to use deep brain stimulation (DBS) to improve behavioral performance in patients with PDOC^7,8^. Most previous studies in humans describe heterogeneous patient populations with different forms of severe brain injury and clinical results of the procedure vary from literally no effects of stimulation to rather spectacular improvements of consciousness^8–11^. In the majority of these cases, the nuclei of the central thalamus, including the centrolateral (CL) and centromedian-parafascicular complex (CM-Pf) were stimulated, based on the hypothesis that DBS of these nuclei may result in re-activation of damaged central thalamic outflow tracts and, therefore, may restore dysfunctional neurocircuits involved in arousal regulation^7,10,12^. The pivotal role of the central thalamus in the regulation of arousal and the possibility of modulating arousal through its fiber pathways using neurostimulation is now demonstrated in an increasing corpus of evidence, mainly derived from animal research^13–17^. Yet, it remains unknown what the exact mechanisms of DBS in humans are responsible for its effects on consciousness and why these effects are so variable^8^.

In the current open-ended exploration, we set out to study the clinical and neurophysiological effects of central thalamic DBS in a single patient with prolonged MCS, more than eight years after brain injury. We conducted an experimental trial of 24 months of DBS targeted at the CM-Pf (see methods), with different stimulation settings, varying in pulse-width and frequency (50 Hz versus 130 Hz), and used magnetoencephalography (MEG) to measure the DBS-induced changes in relative band power, frequency-specific functional connectivity, and neural variability, which are measures used to characterize neuronal activity and functional interactions between brain regions^18^. MEG is a technique that uniquely allows the study of direct stimulation-induced changes in oscillatory activity with an excellent spatial and temporal resolution^19^. The evidence from the MEG recordings is used to propose hypotheses that explain the effects of DBS in patients with PDOC after severe brain injury.

## Results

### Clinical results

At baseline, this patient scored 9–14 on the Coma Recovery Scale-Revised (CRS-R), meaning a baseline MCS- condition. After starting monopolar high-frequency stimulation (130 Hz; 60 μsec) with the more ventral contact points as cathode, situated in the central region of the thalamus, a direct improvement was seen in arousal after reaching a minimum current of 1 mA. These arousal effects included direct pupillary dilation, raising of the otherwise flexed head, increased respiratory rate, and signs of active visual pursuit throughout the room. These arousal effects became increasingly evident when the current was raised to a level of 4 mA. At this level, we observed some signs of purposeful behavior, such as visual interaction with her father (smiling and following him through the room), though these signs remained short-lived (minutes) and could not be reproduced by the clinical investigator/assessor. After various trials of DBS with different contact points, it was decided to set the stimulator on at contact points 3L and 11R, encircling the central nuclei of the thalamus, and to use a cycling mode consisting of 30 min on and 90 min off stimulation. The stimulator was turned on in the morning and off at night by her family. During the following months, the family reported signs of increased instances of visual pursuit during the day, a decrease of spasticity in both arms and legs, and a significant reduction of paroxysmal sympathetic hyperactivity she usually experienced, such as sweating episodes. We decided to wait for the recurrence of signs of awareness and possible signs of ‘upregulation’ of behavior as previously described by other authors^11^. During a period of six months, stimulation parameters were intermittently changed according to observations of behavioral performance by her family. These changes included changes in current, as well as in cycling periods (for study design and stimulation parameters see Supplementary Fig. 1 and Supplementary Table 1). However, after these six months, it was concluded that there were no persistent beneficial changes in behavioral performance with 130 Hz DBS. Therefore, it was decided to switch to monopolar lower frequency stimulation (30 Hz; 450 μsec). After changing the settings, once again, a direct and strong arousal effect was seen, as well as evidence of the return of visual pursuit. Moreover, it was observed that she could perform small tasks on request, such as sticking out her tongue, or moving a finger, though these reactions could not consistently be elicited. A trial episode was started at 2.5 mA on contact points 2 and 3L and 10 and 11R, with a cycling mode consisting of 90 min on and 30 min off. After two months, it was decided to add stimulation during the nights. However, after a couple of days, it was observed that the patient could not sleep at night and kept her eyes open, which caused excessive sleepiness during the day. Thereafter, DBS was discontinued at night and started in the mornings. In the following months, the frequency was increased to 50 Hz and the current was slowly raised to 3 mA with reports of increased arousal, visual pursuit, return of swallowing, and reduction of spasticity. The signs of increased arousal and visual pursuit, such as recognition of family members, were directly seen after starting the stimulation in the morning and only present during moments of stimulation (see Supplementary Video). After discontinuation of stimulation, arousal effects slowly, but progressively vanished, usually within 30 min to 1 h. Therefore, the daytime cycling mode was changed to continuous stimulation. The improvement in swallowing and reduction of spasticity were more permanent and also observed by her speech therapist and physical therapist. Nevertheless, at the end of the trial, no signs of a persistent return of behavioral performance were observed using the CRS-R. The eventual CRS-R at 24 months after implantation remained between 9 and 12, which matches her baseline CRS-R (for subscores, see Supplementary Table 2).

### Neurophysiological results

**Figure 1:**
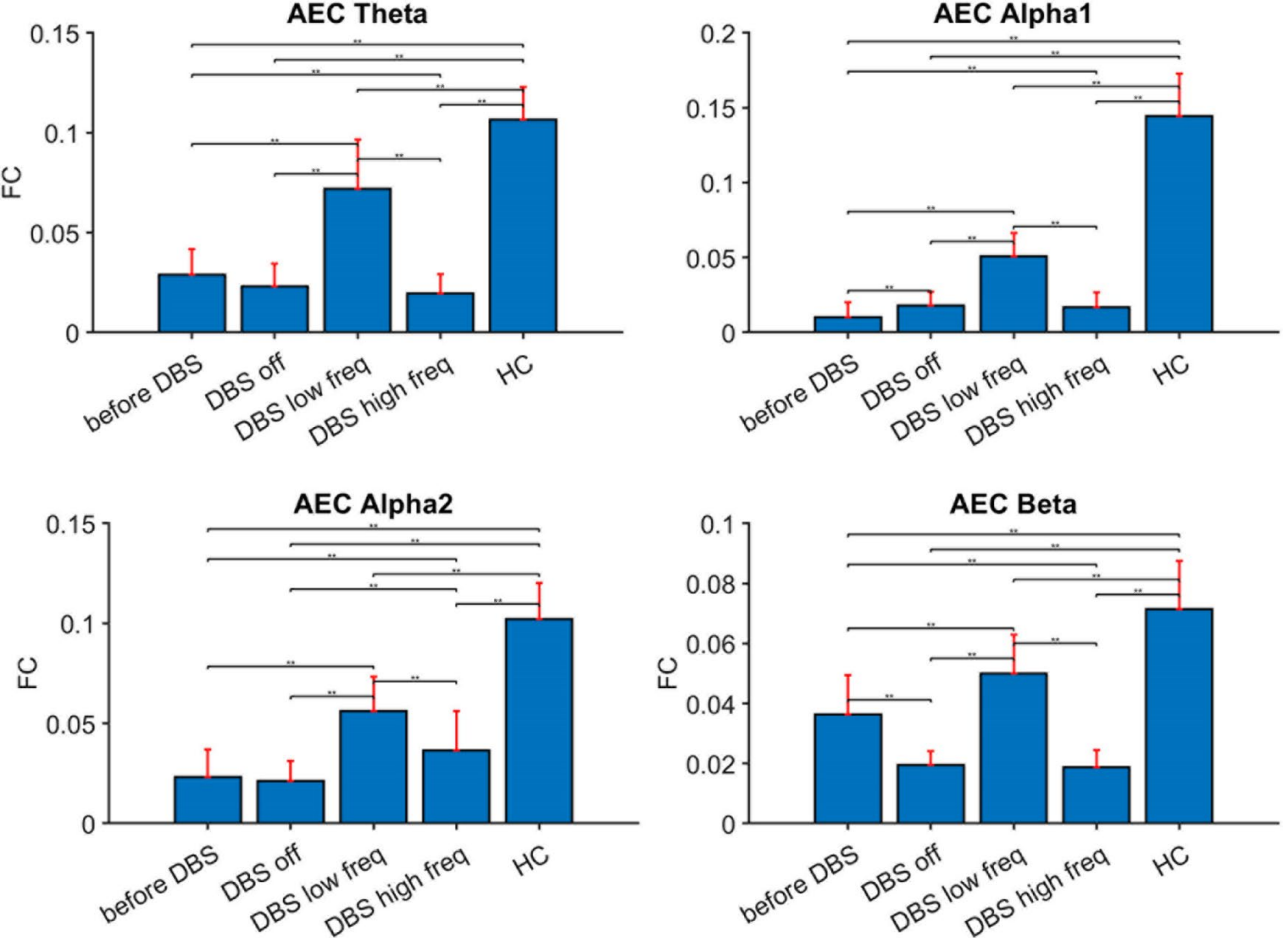
Functional connectivity (FC) in four different conditions in the patient, and for healthy controls (HC). Red bars: median values and interquartile range. Upper left: theta FC; upper right: alpha1 FC; lower left: alpha2 FC; lower right: beta FC. **p < 0.01. AEC = AECc = corrected amplitude envelope correlation, before DBS = before implantation DBS, low freq = deep brain stimulation at 50 Hz/450 μsec, DBS high freq = deep brain stimulation at 130 Hz/60 μsec, DBS off = resting-state before stimulation.

**Figure 2:**
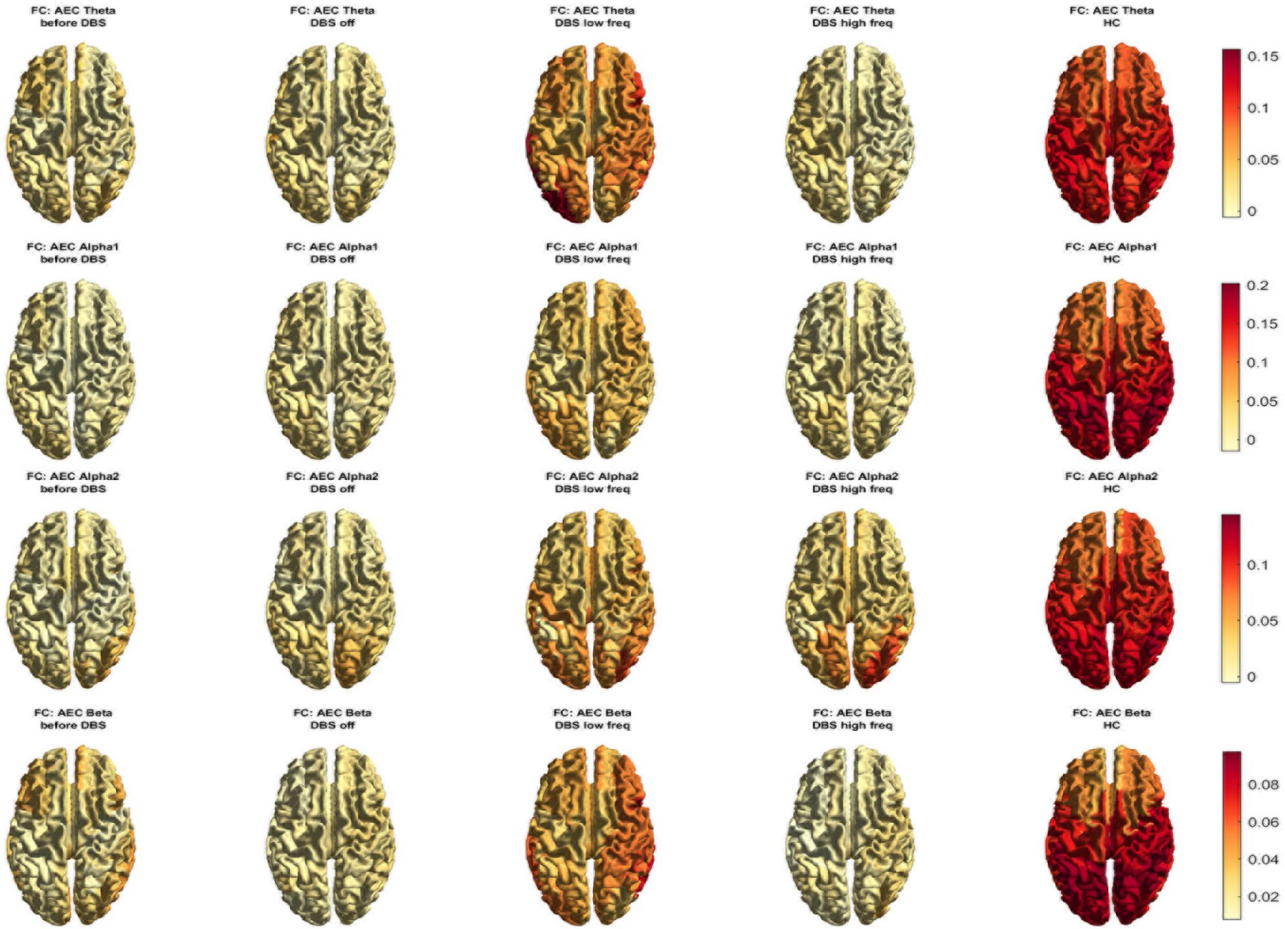
MEG functional connectivity (AECc) for the four different conditions in the patient, and for healthy controls (HC) displayed as a color-coded map on a parcellated template brain, viewed from above. For visualization purposes, only cortical brain regions are displayed. From top to bottom: theta, alpha1, alpha2, and beta-band functional connectivity. Note that different colorbars were used for the different frequency bands. Before DBS = before implantation DBS, DBS low freq = deep brain stimulation at 50 Hz/450 μsec, DBS high freq = deep brain stimulation at 130 Hz/60 μsec, DBS off = resting-state before stimulation.

The MEG source-space spectral analysis of the pre-DBS situation, the post-implantation situation with stimulation turned off (resting-state before stimulation), and active DBS turned on in both lower (50 Hz) and higher frequency (130 Hz) settings revealed no differences in relative band power. In contrast, there were several differences in functional connectivity throughout the brain between the four above-described situations and between the patient and the healthy controls. There was significantly lower functional connectivity in all frequency bands between this patient in the pre-DBS situation and healthy controls (see Figs. 1 and 2, also for *p*-values). 50 Hz stimulation was associated with a significant increase in functional connectivity in all frequency bands, both compared to the DBS off state as well as compared to the period of higher frequency DBS. However, the levels of functional connectivity after 50 Hz stimulation were still significantly lower than those observed in healthy controls. 130 Hz stimulation resulted in a relatively limited increase in alpha2 functional connectivity only, and it remained significantly weaker than following lower frequency stimulation. Moreover, small significant differences in functional connectivity were observed between the pre-DBS and post-DBS off situation, such as a small increase in alpha1 functional connectivity, and a decrease in beta-band functional connectivity in the post-DBS off condition. The above-described differences in functional connectivity were seen throughout the brain with some differences in frontal, parietal, and occipital areas that are variable between different frequency bands (see Fig. 2 and Supplementary Material Fig. 2). Specifically, 50 Hz DBS was associated with some increase of functional connectivity in (especially the right) frontoparietal and occipital areas.

**Figure 3:**
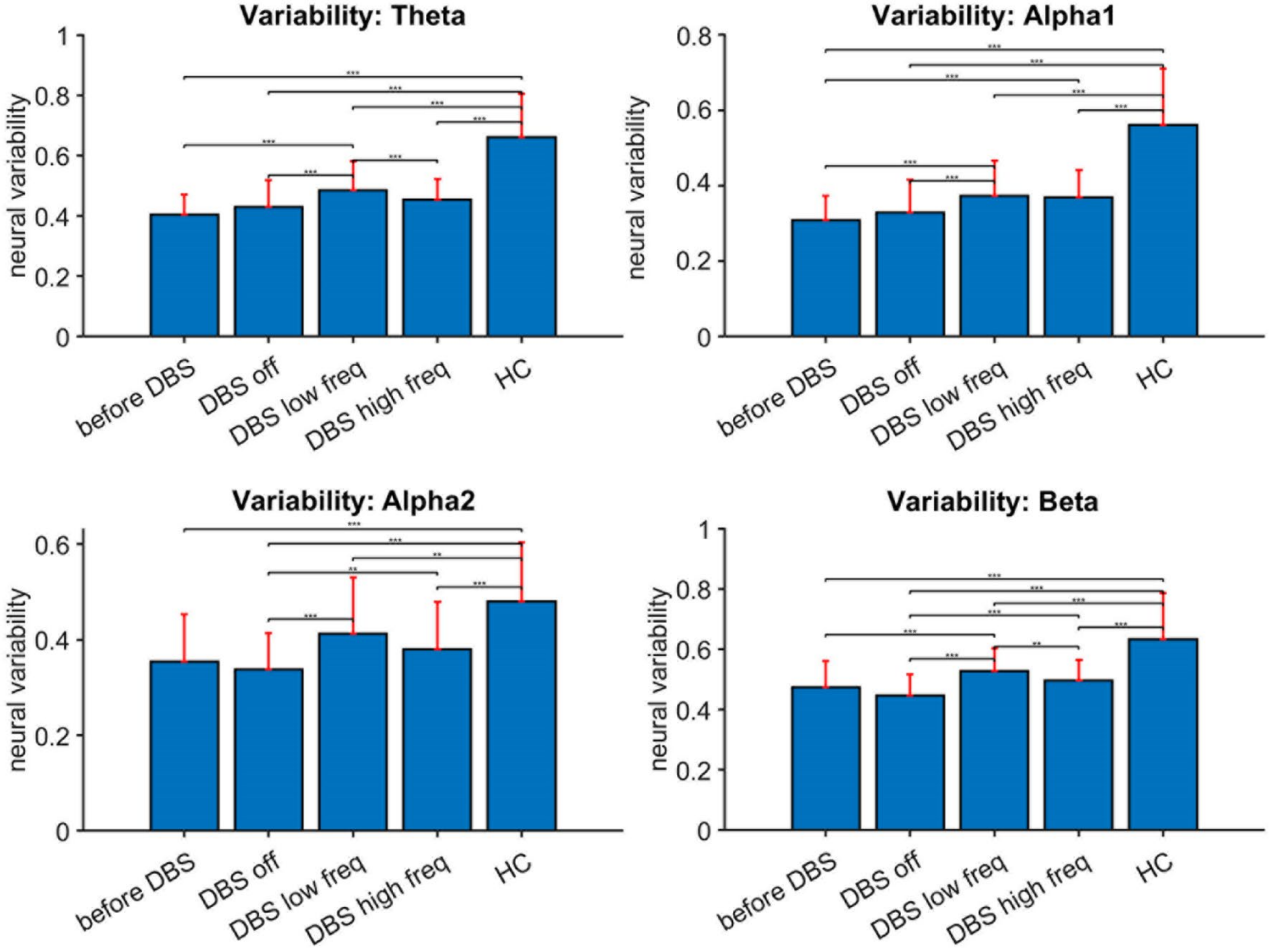
Neural variability for four different conditions in the patient, and for healthy controls (HC). Red bars: median values and interquartile range. Upper left: theta FC; upper right: alpha1 FC; lower left: alpha2 FC; lower right: beta FC. **p < 0.01; ***p < 0.001. Also see Supplementary Material Fig. 3 for regional differences. Before DBS = before implantation DBS, DBS low freq = deep brain stimulation at 50 Hz/450 μsec, DBS high freq = deep brain stimulation at 130 Hz/60 μsec, DBS off = resting-state before stimulation.

We also found significant differences in global neural variability between the different situations and between this patient and healthy controls (see Fig. 3 and Supplementary Material Fig. 3). The patient maintained significantly lower levels of neural variability within the brain in all frequency bands compared to healthy controls. Only 50 Hz stimulation was associated with a small, yet significant, increase in all frequency bands when compared with the other DBS conditions, except when compared with 130 Hz stimulation in the alpha bands, and when compared with the pre-DBS situation in the alpha2 band. 130 Hz stimulation was only associated with a significantly small increase in the alpha2 and beta-band compared to the DBS off state. However, all of these levels were still evidently lower than those observed in healthy controls. The analysis of regional differences between the different conditions showed variable changes in neural variability throughout the brain after stimulation, without a clear consistency in frequency bands to indicate involvement of specific brain areas (Supplementary Material Fig. 3).

## Discussion

### General discussion

In this study, we showed that DBS is associated with significant changes in functional connectivity and neural variability in MCS. DBS with a lower frequency (50 Hz) and larger volume of activation (VTA) was associated with a stronger increase in functional connectivity and neural variability. This increase in functional connectivity and neural variability after DBS was observed in all frequency bands and throughout the brain, suggesting a widespread reorganization of brain networks, though these neurophysiological markers remained significantly lower after DBS than observed in healthy controls. The increase in functional connectivity and neural variability was paralleled by direct increases in arousal and more subtle permanent improvements in visual pursuit, spasticity, and swallowing, though, no improvements in behavioral performance were observed. The return of arousal and some basic functions without an overall improvement of behavioral performance may indicate that some disrupted neural networks are re-activated by DBS, but that the injured brain still lacks the ability to adapt to changing cognitive demands. It may be indicative of the severity of the brain injury and represent a state in which the brain is unable to work as a coherent unit.

Some observations from this study correlate with other studies in patients with PDOC. We showed that functional connectivity in the resting-state of our patient was significantly lower for all frequency bands compared to resting-state examinations in healthy subjects. It is known that levels of functional connectivity are, more or less, inversely correlated with the severity of impairments in consciousness. For instance, UWS patients are known to have significantly lower levels of functional connectivity throughout the brain than those with MCS and patients recovering from severe brain injury show increasing levels of functional connectivity throughout their period of recovery^20–22^. Previous studies with patients with PDOC have shown decreased levels of functional connectivity in specific brain networks, especially in frontoparietal networks, subcortical networks, and the default mode network, all believed to be involved in arousal regulation^23^. However, in our analysis, there were only small regional differences in functional connectivity. Similar observations have been reported for neural variability. For instance, previous research has shown a loss of variability after severe brain injury^24,25^. Neural variability is a measure of the ability of the brain to adapt to rapid changes in cognitive demands^26^. It has been shown to be a much better correlate for behavior than traditional measures of neuronal oscillatory power^27,28^. Awareness is thought to depend strongly on neural variability of large-scale cortico-thalamo-cortical networks^29,30^. The observation that, after DBS, neural variability remains lower than in healthy controls may therefore indicate that, despite improvements in arousal, awareness is still significantly disturbed. The heterogeneity of brain damage possibly causes different levels of network disruption resulting in different baseline levels of functional connectivity and neural variability in each individual patient^31^. This may mean that some patients may be more susceptible to the effects of thalamic DBS than others. It remains a matter of future research to define if pre-DBS levels of functional connectivity and neural variability may be used as biomarkers for a patient’s responsiveness to any neuromodulatory intervention.

### Limitations

Our findings should be interpreted with some caution. Firstly, different stimulation parameters were used in our study and were based on previous clinical experience in other neurological conditions and clinical (side) effects during the titration phase of the study. The use of a different pulse width and frequency results in different effective electrical field sizes and corresponding VTA within the central thalamus (see Supplementary Material Fig. 4). Consequently, using different stimulation parameters, a different mix of (central) thalamic nuclei could have been stimulated, especially in a severely atrophic brain with a shrunken thalamus. Similar situations have been described in animal DBS research, in which a smaller thalamus, more densely packed with nuclei, is possibly more affected by nonspecific spreading of DBS currents near the surroundings of the intended target area^17^. The reconstruction of the VTA’s of both stimulation frequencies shows a rather large area of stimulation, likely including both the CM-Pf, CL, and parts of other intrathalamic pathways that are involved in arousal regulation, such as the medial dorsal tegmental tract (see Supplementary Material Fig. 4 for a 3D overview of both VTA’s)^16^. The reconstruction also shows that the 50 Hz DBS regime, with a larger pulse-width, did not only extend more laterally, but also more ventrally than the 130 Hz DBS regime This could also allow for concomitant anti-dromic modulation of different brainstem nuclei that project into the intralaminar nuclei which are known to be involved in arousal regulation, including those from, for instance, the pedunculopontine arousal system that have a frequency effectiveness plateau around 40–60 Hz^32^. Thus, although we intentionally targeted the DBS electrodes to the CM-Pf of the central thalamus using traditional stereotactic coordinates, the mechanism of action of the observed arousal and autonomic response and concomitant changes in functional connectivity/variability may also directly or indirectly involve areas well beyond the central thalamus itself. The differences that are observed between lower and higher frequency stimulation are somewhat surprising since previous DBS studies in both animals and humans mainly used stimulation at higher frequencies to increase arousal and even reported signs of behavioral arrest after (very) low-frequency stimulation^7,8,33^. This may also mean that the effects of stimulation are patient-specific and/or that the effects of neurostimulation differ between animals and humans. Secondly, this study is subject to performance confounding, meaning that if the behavioral performance is substantially different in two experimental conditions, as is the case for the pre- and post-DBS conditions, some measures of brain physiology might also differ between those conditions, thereby representing an epiphenomenon^21,34^. Neurophysiological activity is well known to change under different behavioral conditions. For instance, the changes in the alpha-band associated with transitions between arousal states observed in the present study are consistent with the functional roles of alpha-band oscillations in the regulation of attention and information processing^35^. Thirdly, during 50 Hz stimulation, there were significant artifacts in the MEG signal, limiting its direct analysis. Therefore, for the analysis of 50 Hz stimulation, we were obliged to perform the analyses directly after cessation of the stimulation. These artifacts of DBS are well known in MEG research^36^. Previous studies showed that the effects of stimulation can be reliably analyzed after cessation, and, because the effects of stimulation on arousal were relatively long-lasting (> 30 min after stimulation), we think that this potential limitation is negligible^36^. Fourthly, arousal in patients with MCS varies during the day, which may complicate the comparison between conditions. These variations, including the 15 months interval between baseline and experimental MEG recordings, may contribute to the slight differences between the pre-DBS and post-DBS off-state in this patient. Lastly, statistics with n = 1 remain challenging and, since amplitude envelope data is non-Gaussian, we were restricted to non-parametric tests to evaluate differences between conditions. In addition, recordings were too short to perform robust trial-by-trial statistics. Hence, current statistical results should be interpreted with caution.

### Ethical considerations

The decision to carry out research and perform an experimental neurosurgical procedure in a mentally incompetent patient was not made lightly. We specifically selected a subject in MCS instead of UWS, because MCS patients are known to have more intact, but ‘dormant’ cerebral networks that may be more susceptible to the effects of activation by neurostimulation^10,37^. Previously, several ethical reasons have been proposed to continue treatment and the pursuit of higher levels of consciousness in patients with MCS that are known to have covert cognitive capabilities, but remain in affective and cognitive isolation^10,38,39^. One of the most important purposes of DBS in this study was to improve the patient’s ability to interact with others in a meaningful manner, providing an opportunity for a more detailed assessment of her feelings and preferences and permitting the patient to assume a more active role in her treatment^10^. The patient’s family were the legal representatives of the patient and were actively involved during the study, accompanying her with hospital visits, helping us in the evaluation of the effects of the DBS, and monitoring for possible signs of discomfort. To minimize burdensome travels for the patient and her family, follow-up took place at her home with both operating surgeons paying her multiple visits during the DBS titration phase. Though the primary goal of this study, improvement of behavioral performance, was not reached, there were improvements in her condition that were eventually considered meaningful by her family, including reduced spasticity, fewer periods of paroxysmal hyperactivity, and improvement of swallowing, which even allowed the patient to eat by mouth. These improvements were considered a benefit for the patient, since they seemed to reduce suffering. In the current patient, DBS was performed eight years after brain injury. One could hypothesize that performing DBS at an earlier stage could possibly have had more beneficial effects, for instance by reducing long-term complications of disorders of consciousness, such as spasticity, rigidity, contractures, and severe tongue/muscle atrophy.

## Conclusion

In conclusion, this study shows that DBS can re-activate ‘dormant’ functional brain networks, but that the severely injured brain still lacks the ability to serve cognitive demands. This evidence provides more fundamental insight into the network-level mechanisms underlying DBS of the intralaminar thalamus and inspires future research on neurostimulation in patients with PDOC, especially on DBS in those patients who have more intact brain networks and residual dynamic properties of functional connectivity. A larger sample of patients is necessary to build further on our preliminary findings. International collaboration and clustering of cases in specialized centers will be vital to explore the possible application of DBS to restore function in this group of severely impaired patients.

## Methods

### Ethical approval

The legal representatives of the patient gave written informed consent to the research protocol, which was approved by the medical ethical committee of the Amsterdam University Medical Centers (protocol number: NL58841.018.16). Moreover, written informed consent was obtained for publishing information/images/videos in an online open-access publication. Ethics review criteria conformed to the Declaration of Helsinki. No part of the study procedures or analysis plans was pre-registered in an institutional registry prior to the research being conducted.

### Clinical case description

A 38-year-old female without any relevant medical history sustained severe traumatic brain injury in a road traffic accident, eight years prior to this study. The initial Glasgow Coma Scale score was E1M4V2, after which she was intubated and transferred to the hospital. A CT-scan of the head revealed a left-sided occipital condylar fracture and multiple contusions in the dorsal mesencephalon, right basal ganglia, left thalamus, and periventricular areas. In addition, she sustained multiple bone fractures. Since there were no signs of significant mass effect or brain herniation, she only received an intracranial pressure monitor. MRI after one day showed signs of diffuse axonal injury (Adams grade III), including hemorrhagic components in the corpus callosum and cerebellar peduncles. She was sedated with propofol for several days in the Intensive Care Unit to reduce intracranial pressure. Intracranial pressure measurements remained low (< 20 mm Hg). After cessation of sedation, the patient remained behaviorally unresponsive. Her best GCS-motor score remained 4 during the initial hospital admission. In the intensive care, she developed seizures, for which she was treated with anti-epileptic drugs, as well as episodes of paroxysmal sympathetic hyperactivity, for which she received clonidine and baclofen. She received a tracheostomy and jejunostomy, and after approximately one month, the patient was transferred to another hospital with a preliminary diagnosis of an unresponsive wakefulness syndrome (UWS), previously called vegetative state. After this short secondary hospitalization, she was transferred to a comprehensive inpatient brain injury rehabilitation program where physical, speech and related therapies were performed. During this inpatient rehabilitation course, her level of arousal improved and some evidence of following commands and short periods of coherent speech was reported by her family. However, her condition regressed to a state wherein only inconsistent command-following and pursuing eye movements were noted, indicating transition into MCS. She remained nonverbal and developed severe spasticity involving all four extremities. For the next seven years she received home care at her parental home. However, there were multiple acute care/hospital admissions for recurring pulmonary infections. Approximately eight years after the initial injury, the patient was re-admitted to our hospital for assessment of her clinical condition and enrollment in the current study.

On examination, the patient was alert and demonstrated spontaneous, though inconsistent, signs of visual fixation. The right pupil was minimally reactive and the left pupil was fixed. The oculomotor exam demonstrated disconjugate eye movements. Reflexes were hyperactive throughout. Some signs of sympathetic hyperactivity were still present, including excessive sweating, drooling, and recurrent episodes of tachypnea. The motor exam was notable for severe spastic contractures of all four extremities with flexion at elbows, wrists, fingers, and knees. There were also equinovarus deformities involving both feet. The axial tone of neck musculature was severely reduced with a continuous flexion position of her neck. Command-following was inconsistent with some minor evidence of reactions to verbal and visual cues (turning her head towards voices or her family), although there was no adequate response to complex commands. She could not speak, nor communicate reliably with yes/no responses, though, at times, could stick out her tongue on request or imitation. EEG examination revealed no signs of epilepsy, with a background pattern with relatively little differentiation, predominantly fast activity, and signs of reactivity with eye-opening. A structural MRI revealed widespread cortical atrophy, including marked tissue loss in both frontal regions, as well as in the basal ganglia, thalamus, and mesencephalon. Medications during the study period remained unchanged and included amantadine and baclofen, drugs that are often given to patients with decreased levels of consciousness to reduce spasticity and increase daytime arousal.

### Clinical assessments

The current explorative study was designed to evaluate the clinical and neurophysiological effects of DBS of the central thalamus in a patient in MCS for the duration of 24 months (see Supplementary Material Fig. 1 for study design). After inclusion in the study, one month before DBS implantation, four separate baseline assessments of the level of consciousness were done using the Coma Recovery Scale-Revised (CRS-R). The CRS-R is the most recommended assessment scale for level of consciousness determination in PDOC patients^40–42^. It is a compound scale covering the domains of auditory, visual, motor, and verbal function, responsiveness, and arousal. The total score ranges from 0 (worst) to 23 (best) with specific subscores that can individually denote MCS-, MCS + , or emergence from MCS. Since the CRS-R has a ceiling effect, video recordings were performed to qualitatively capture the full spectrum of behavioral changes. The CRS-R examinations and video recordings were performed in the patient’s parental home to avoid unnecessary hospital visits. At the end of the study, 24 months after DBS implantation, four additional CRS-R examinations and video recordings were made to assess the clinical changes after long-term stimulation.

### Surgical strategy: implantation of DBS electrodes

**Figure 4:**
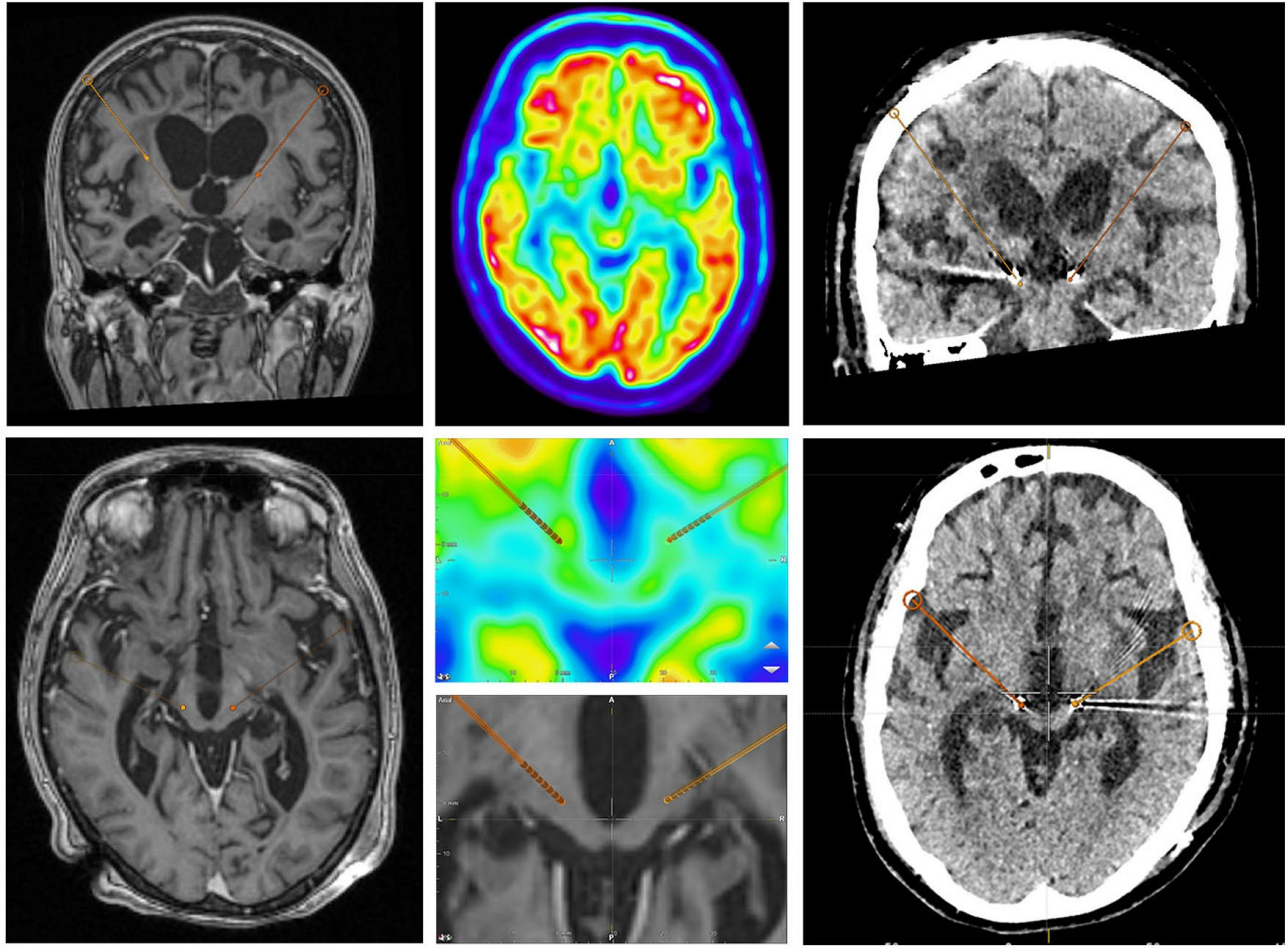
Left panel: intra-operative planning MRI with trajectories. Middle panel: ^18^F-FDG PET-scan of target areas, including planned trajectories. Right panel: post-operative CT-scan with electrode localization.

Before surgery, the patient underwent a 3T stereotactic MRI (Siemens, Malvern, Pennsylvania, USA), including axial T2-weighted and post-gadolinium (Gd) volumetric axial T1-weighted sequences. Pre-operative CM-Pf targets were determined from the mid-commissural point on anterior–posterior (AC-PC) aligned MRI images. Target planning for the central thalamus (intentionally aimed at the CM-Pf complex using traditional stereotactic methods) was optimized, based on the width of the third ventricle with final coordinates: 9.8 mm lateral, 9.5 mm posterior, and 2.8 mm ventral to the midcommissural point. Planned trajectories were inspected to be pre-coronal, start on top of a gyrus, and to avoid ventricles and blood vessels. ^18^F-fluorodeoxyglucose (FDG)-PET/CT brain imaging was performed on a Siemens PET/CT system (Biograph mCT FlowTrue-V-128), conform European Association of Nuclear Medicine guidelines, to confirm the presence of FDG-uptake in the center of the anticipated target area (see Fig. 4)^43^. More specifically, FDG images were acquired for 10 min, starting at 30 min after bolus intravenous injection, and low-dose CT was used for attenuation correction. Images were reconstructed iteratively with point‐spread function and time‐of‐flight modelling, and a 2‐mm full‐width at half‐maximum Gaussian filter. Planning, including fusion of the MRI and PET was done using Brainlab Elements software (Brainlab AG, Munich, Germany, version 3.2.0.281).

On the day of the surgery, a Leksell stereotactic frame (G-model, Elekta Ab, Stockholm, Sweden) was placed under general anesthesia and the patient was transported to the 1.5T MRI, where a frame-based stereotactic MRI was obtained. After fusion with the pre-operative 3T MRI, stereotactic coordinates of the planned targets were obtained. The patient was returned to the operating room and burr holes were made. A rigid macrostimulation electrode (Elekta) was inserted into the left and right target and replaced by a Boston Vercise™ Cartesia lead with eight 1.5 mm contact points separated by 0.5 mm interspaces (model DB2202, Boston Scientific, California, USA) under fluoroscopy. Left-sided ventral-to-dorsal contacts encoded 1–8 and right-sided ventral-to-dorsal contacts encoded 9–15. Subsequent implantation of a corresponding Boston Vercise™ pulse generator was done in a subcutaneous pocket in the infraclavicular region under general anesthesia in the same surgical session. One day after the operation, a CT-scan was made and co-registered to the MRI for lead localization. The patient was discharged two days after surgery.

### Clinical follow-up and DBS ‘titration’

Two weeks after discharge, the pulse generator was turned on. During this experimental ‘titration-session’, a wide variety of settings was used to carefully study the direct effects of DBS on arousal, and to rule out any discomfort or aberrant neurological symptoms. Similar to DBS in movement disorders, programming was started with monopolar high-frequency stimulation (130 Hz; 60 μsec). Thereafter, the patient was visited multiple times and slight adjustments in DBS settings were made to test the effects on arousal and rule out any discomfort or aberrant neurological symptoms (for an overview see section Clinical Results and Supplementary Table 1). Changes in behavioral performance were directly evaluated. Additional visits were made at the request of the family when signs of behavioral responsiveness or deterioration were noted throughout the study period. After one year of high-frequency stimulation, a switch was made to a lower frequency setting (30 Hz; 450 μsec, similar to periaqueductal gray DBS in chronic pain), since a significant change in behavioral performance remained absent. Once again, different stimulation parameters were studied (see Supplementary Table 1). Finally, a switch to monopolar 50 Hz stimulation was made, which, after clinical evaluation, was considered the optimal setting (50 Hz; 450 μsec; 2.5 mA). Fifteen months after DBS implantation, and after using the various stimulation parameters, MEG studies were performed to evaluate the neurophysiological profiles of both stimulation settings. The effective electrical field size of both DBS regimes was visualized by calculating the volume of tissue activated (VTA) in Brainlab’s Guide-XT software module (Brainlab AG, Munich, Germany, version 3.2.0.281) (see Supplementary Material Fig. 4).

### MEG data-acquisition and pre-processing

The MEG recordings were obtained in a magnetically shielded room in a supine resting-state condition. Pre-DBS, 30 min of MEG data had been recorded as a reference for further research. Fifteen months after implantation, multiple post-DBS datasets were recorded, starting with another resting-state condition without stimulation (the “DBS off” condition). The stimulator had been off for 12 h. Hereafter, the DBS was turned on, starting with low-frequency (50 Hz; 450 μsec; 2.5 mA) stimulation. After 10 min of recording, the DBS was turned off and a wash-out period followed (five minutes), which was also recorded since 50 Hz stimulation causes artifacts in MEG signal. Then the patient was removed from the magnetically shielded room for more than an hour, which was considered a normal wash-out period for all arousal effects of low-frequency stimulation. The DBS was then changed to the higher frequency setting (130 Hz; 60 μsec; 2.5 mA) and another 10-min MEG recording followed, succeeded by another wash-out recording of five minutes.

MEG data were recorded using a 306-channel whole-head system (Elekta Neuromag Oy, Helsinki, Finland) with a sampling frequency of 1250 Hz and online anti-aliasing (410 Hz) and high-pass filtering (0.1 Hz). The head position relative to the MEG sensors was recorded continuously using the signals from five head position indicator (HPI) coils. The HPI positions and the outline of the patient's scalp (around 500 points) were digitized before the MEG recording using a 3D digitizer (Fastrak, Polhemus, Colchester, VT, USA). The patient's MEG data were co-registered to her structural MRI, using a surface-matching procedure with an estimated resulting accuracy of 4 mm^44^. This structural MRI of the head had been obtained two months before the baseline MEG session as part of clinical care, using a 3T Siemens MRI scanner (Siemens, Malvern, Pennsylvania, USA).

For MEG source-level analysis, extra processing steps were undertaken. The MEG data were first cleaned using both spatial and temporal filtering, after which the sensor-level data were projected to source-space using an atlas-based beamformer. Neuronal activity (relative power and variability) and functional connectivity were quantified at the source-level. In more detail, bad channels and data segments were first removed after visual inspection of the data. Thereafter, the temporal extension of Signal Space Separation (tSSS) in MaxFilter software (Elekta Neuromag Oy, version 2.2.15) was applied using standard settings: a correlation limit of 0.9, and a sliding window of 10 s^45,46^. The automated anatomical labeling (AAL) atlas was used to label the voxels in 78 cortical and 12 subcortical regions of interest (ROIs)^47,48^. This was done by registering the anatomical T1-weighted image to an MNI template and labeling all voxels according to the 90 ROIs. Subsequently, an inverse registration to anatomical subject space was performed. We used each ROI's centroid voxel as a representative for that ROI^49^. Subsequently, a scalar beamforming approach (beamformer, version 2.1.28; Elekta Neuromag Oy), similar to Synthetic Aperture Magnetometry (Robinson and Vrba, 1999) was used to project the sensor-level data to these centroids. The beamformer weights were based on the covariance of the recorded time-series within a 0.5–48 Hz frequency window and the forward solution (lead field) of a dipolar source at the centroid voxel location, and using a single sphere head model fitted to the MRI scalp surface as extracted from the co-registered MRI^50^. Source orientation that maximized the beamformer output was obtained using eigenvalue decomposition^51^. Singular value truncation was used when inverting the data covariance matrix to deal with the rank deficiency of the data after SSS (∼ 70 components). Broadband data (0.5–48 Hz) were projected through the normalized beamformer weights^52^, resulting in a broadband time series for each centroid of the 90 ROIs^49^.

The amount of data used for further analysis was determined by the amount of artifact-free data (based on visual inspection by PT) for any of the four conditions (pre-DBS, DBS off, DBS low-frequency stimulation, DBS high-frequency stimulation). Based on this, we kept the amount of data the same for all conditions (4.5 min). For analysis of low-frequency stimulation, we used the MEG dataset during the washout phase, the first minutes directly after cessation of stimulation, since low-frequency stimulation was associated with a direct MEG-artefact, which limited its interpretation. MEG-based functional connectivity (see below) for the patient (in all conditions) was compared with the average functional connectivity obtained from healthy volunteers. Based on gender and age, we selected out of a previously published dataset of healthy volunteers all females of approximately the same age as this patient^49,53^. This resulted in six healthy age-matched females (mean age of 39), who had all undergone one five-minute, eyes-closed, resting-state MEG recording. Data acquisition, pre-processing, and analysis was performed in the same way as for the patient dataset.

### MEG: estimation of functional connectivity and neural variability

We estimated power spectral densities using a Fast Fourier Transform (FFT) after applying a Hanning window. To this end, we used overlapping epochs of 3.2 s (4096 samples). Power spectral densities were averaged over all ROIs and epochs. We defined frequency bands as follows: theta (4–8 Hz), alpha1 (8–10 Hz), alpha2 (10–13 Hz), and beta (15–25 Hz). The choice for this range for the beta band was justified by the presence of artifacts in the data with frequency components above 25 Hz for recordings during which DBS was on. Functional connectivity was estimated using the amplitude envelope correlation (AEC) from band-pass filtered time-series^54^. The AEC captures co-fluctuations in modulations of the amplitude envelope. Pairwise orthogonalization was first performed to reduce the effects of signal leakage prior to connectivity estimation^55,56^. The amplitude envelopes were extracted from the analytical signal obtained from the Hilbert transform for every band-pass filtered time series. No further smoothing or downsampling was applied to the amplitude envelopes. Input to the AEC computation was the total amount of artifact free data (4.5 min for all conditions, see above), hence data was not fed in short epochs to the AEC computation. Pearson correlations between amplitudes envelopes were computed The implementation of the AEC was the same as in Brookes et al. 2016^57^. The AEC was calculated for all possible pairs of ROIs, resulting in a 90 × 90 weighted adjacency matrix that contained the functional connectivity values between all pairs (with a potential range of values between − 1 and 1). Averaging over rows in this weighted adjacency matrix subsequently led to one mean functional connectivity value per ROI (i.e. the average functional connectivity of that ROI with the rest of the brain) per condition. Further averaging across mean connectivity values per ROI resulted in the global (whole-brain) functional connectivity for that condition.

Past work has shown that fluctuations in the amplitude envelope coincide with strong functional connectivity, i.e. periods of high amplitude envelope could serve as a window of opportunity for ongoing functional connectivity^58^. Hence, as the AEC captures co-fluctuations in the amplitude envelope, we lastly estimated the variability of the amplitude envelopes in the context of neural variability^26^. Neural variability was quantified in terms of the detrended standard deviation of the amplitude envelope for every ROI. We first subtracted the mean from every time series for every ROI, after which we computed the standard deviation (based on the total amount of artifact free data without dividing the data into epochs)^59^. The lower limit for values for detrended standard deviation is zero and the upper limit is determined by the range of the values for every time series. Power spectral density, neural variability, and functional connectivity analyses were performed in MATLAB 2018b (Mathworks; 9.1.0.441655) using in-house scripts.

### Statistics

We compared the mean AEC between the different conditions (average over rows and columns in the AEC matrix). Since values between conditions were dependent and non-Gaussian we used a non-parametric permutation test to assess differences between the means^60^. Note that we obtained one AEC matrix per condition. The genuine difference in mean AEC between two conditions was compared to a null distribution of mean differences of surrogate data. A null distribution was obtained as follows: (*i*) starting points were two AEC matrices from condition one (AEC_1_) and two (AEC_2_); (*ii)* we then created two dummy matrices A and B. We assigned a value to an entry *i,j* in the dummy matrix A by randomly selecting a value from either AEC_1_*(i,j)* or AEC_2_*(i,j)*. Here, *i, j* were the same for dummy matrix A and AEC_1_ and AEC_2_. This was done for all matrix entries of dummy matrix A; (*iii)* the entries from AEC_1_*(i,j)* or AEC_2_*(i,j)* that were not selected for dummy matrix A were used to create dummy matrix B. Again, matrix elements *i,j* in dummy matrix B were only assigned by matrix values from AEC_1_*(i,j)* or AEC_2_*(i,j)* with the same index *i,j*; (*iv)* we computed the mean for every dummy group (average over rows and columns of A or B) and their difference was added to the null distribution; (*v)* this procedure was repeated 100,000 times to obtain a null-distribution of mean differences. The genuine difference between the mean values was assumed to be significant if this value was in the right 2.5% tails of the distribution. The same test was used to test a difference between healthy controls and subject-specific condition, with the difference that we used the average AEC matrix across healthy controls as input rather than individual matrices. The same test was also used for neural variability, with the difference that we created a null distribution based on the mean difference of two dummy vectors and not dummy matrices. We performed correction for multiple tests using the False Discovery Rate (80 tests: 2 (number of metrics) × $$\left(\begin{array}{c}5\\ 2\end{array}\right)$$ (number of comparisons between conditions/groups) × 4 (number of frequency bands)).

## Supplementary Information

Supplementary Information 1.

Supplementary Video 1.

## Data Availability

The MEG datasets used and/or analysed during the current study are available from the corresponding author on reasonable request.
