# Supplementary material for: Clinical and neurophysiological effects of central thalamic deep brain stimulation in the minimally conscious state after severe brain injury
Source: Sci Rep. 2022 Jul 28;12:12932. doi: 10.1038/s41598-022-16470-2 (PMC9334292; doi:10.1038/s41598-022-16470-2)
Supplement: Supplementary file 1 — Supplementary Information 1. [file 41598_2022_16470_MOESM1_ESM.pdf]

SUPPLEMENTARY MATERIAL

Supplementary figure 1. Study design.

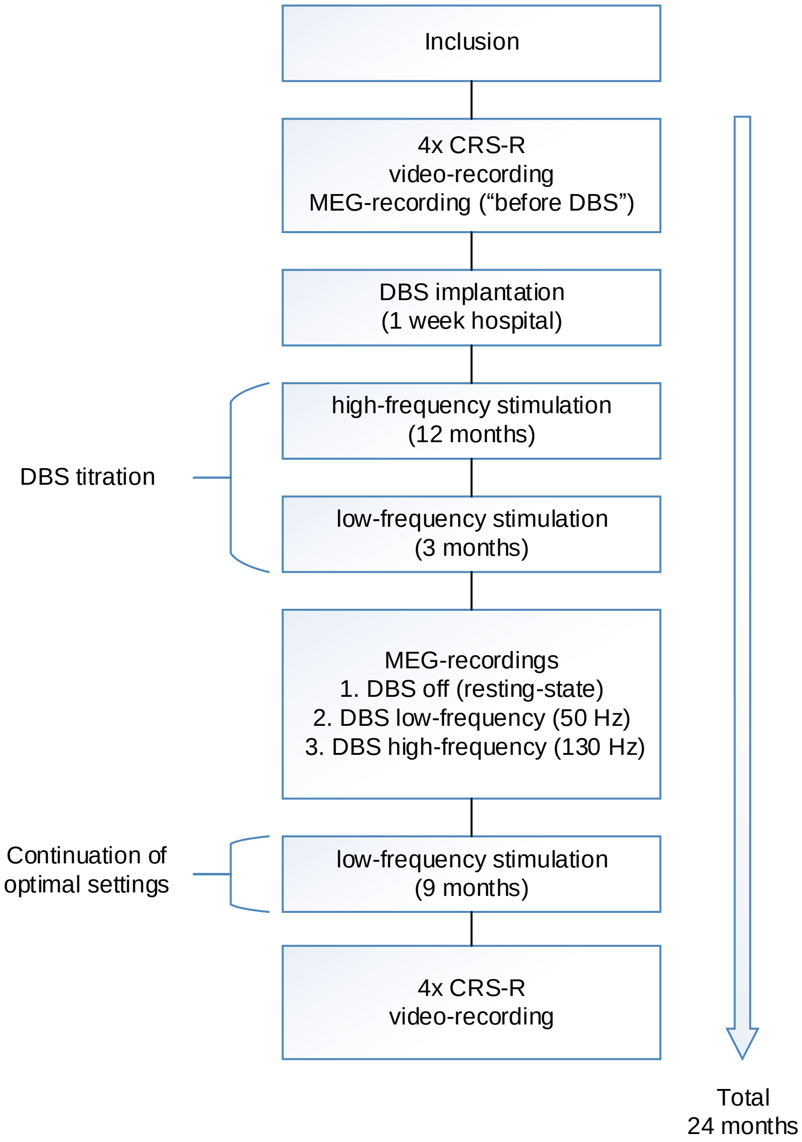

Supplementary figure 2. Reconstructed functional connectivity matrix with the connections for each region of interest for the different frequency bands (for regions see supplementary table 3). Blue = regions with low or negative connectivity, yellow = regions with high-connectivity. From top to bottom: theta, alpha1, alpha2, and beta-band. Note the appearance of four blocks in the matrices for several frequency bands. These blocks are formed by connections between parietal-occipital regions. Note that different color bars were used for the different frequency bands. Before DBS = before implantation, DBS on low freq = deep brain stimulation at 50 Hz/450 µsec, DBS on high freq = deep brain stimulation at 130 Hz/60 µsec, DBS off = resting-state before stimulation.

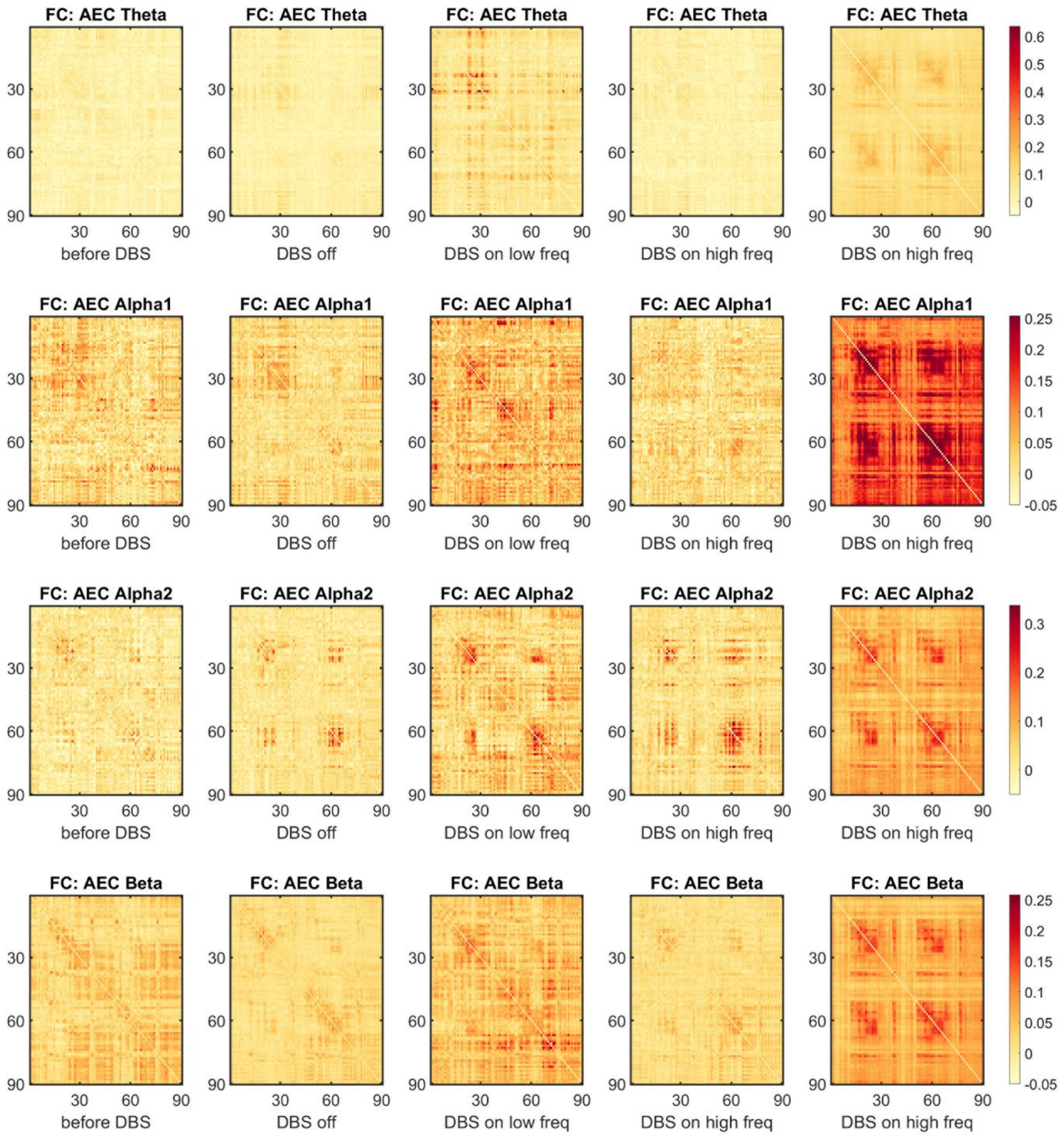

Supplementary figure 3. Neural variability for the four different conditions compared to healthy controls (HC) displayed on a parcellated template brain, viewed from above. For visualization purposes, only cortical brain regions are displayed. From top to bottom: theta, alpha1, alpha2, and beta-band neural variability. Note that different color bars were used for the different frequency bands. Before DBS = before implantation, DBS low freq = deep brain stimulation at 50 Hz/450 µsec, DBS high freq = deep brain stimulation at 130 Hz/60 µsec, DBS off = resting-state before stimulation.

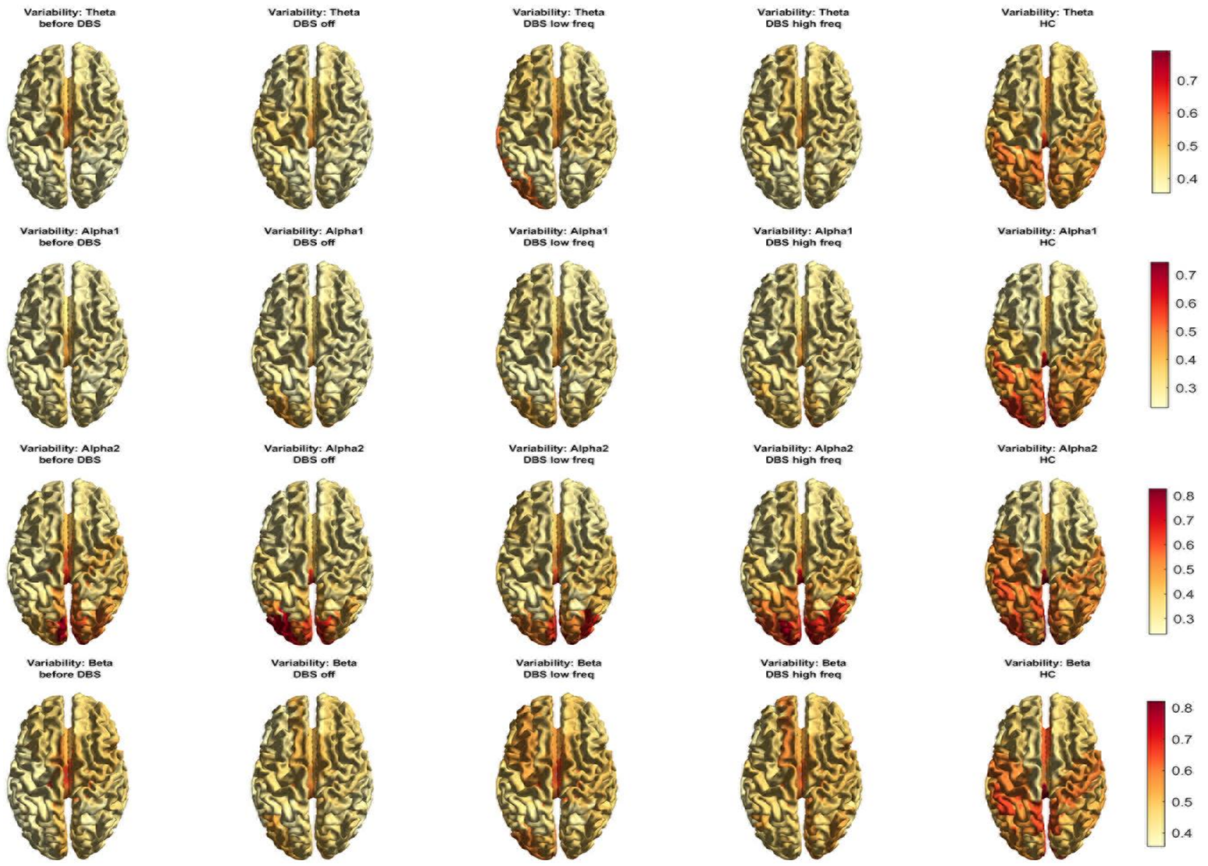

Supplementary figure 4. Effective electrical field sizes of low- (50 Hz) and high-frequency (130 Hz) DBS reconstructed using Brainlab's Guide-XT software and automated postoperative electrode localization. Left panel and upper right panel: axial MRI slice showing the Boston Scientific DB2202 electrode with 8 contact points localized in the central thalamus with the CM-Pf as target. The purple circle represents the volume of tissue activated (VTA) of low-frequency stimulation (50 Hz; 450 μsec; 2.5 mA) on contact points 2 and 3L and 10 and 11R. The blue circle represents the (smaller) VTA of high-frequency stimulation (130 Hz; 60 μsec; 2.5 mA) of the same contact points. Lower right panel: 3D-automated reconstruction of both VTA's.

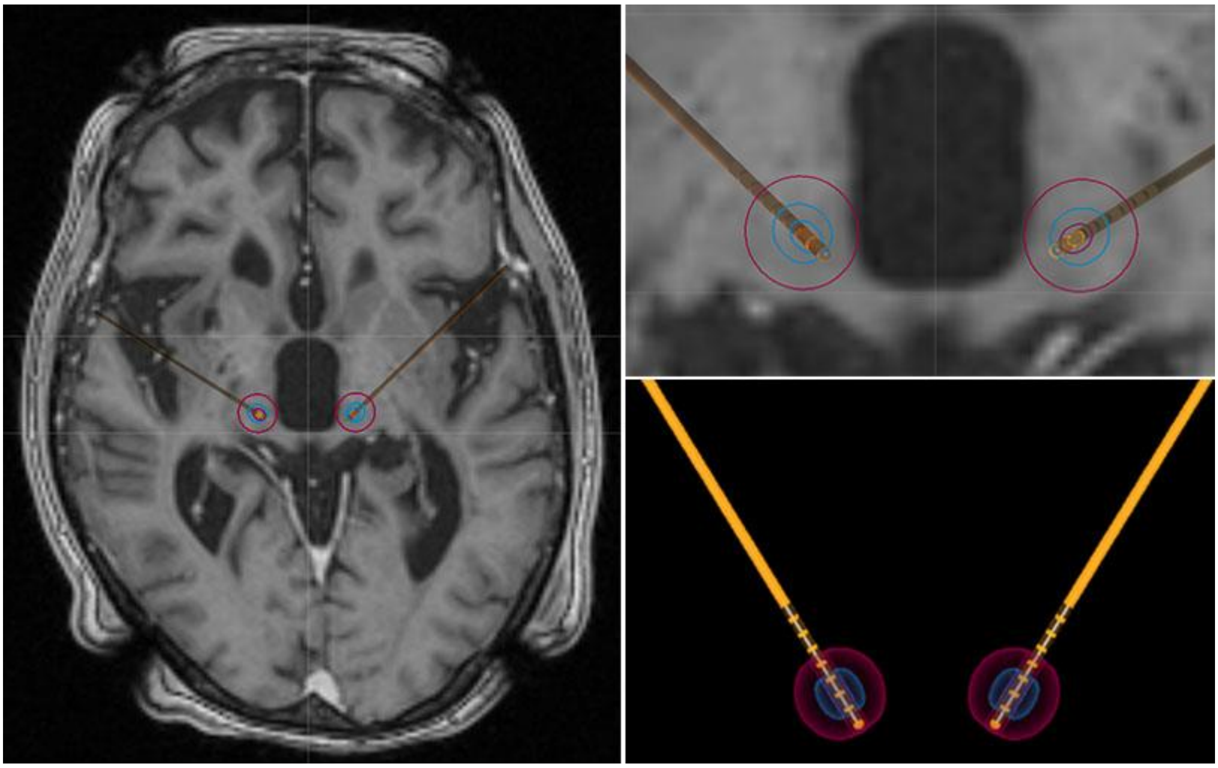

Supplementary table 1. Programming schedule ('titration')

**Phase of study**	**Programming parameters**
Start	130 Hz; 60 µs; 4 mA; cycling-mode (30 minutes on/90 minutes off); Off at night; contact points 3L and 11R.
1 month	Change in cycling-mode (60 minutes ON/60 minutes off).
1.5 months	Change in cycling mode (90 minutes on/30 minutes off).
2 months	Turning cycling-mode off. Stimulator on at daytime and still off at nighttime.
6 months	Switch to 30 Hz; 450 µs; 2.5 mA; Off at night; contact points 2 and 3L and 10 and 11R.
8 months	Change to contact points 6 and 7L and 14 and 15R. Stimulation day and night on (eventually discontinued stimulation during the night).
10 months	Change to contact points 2 and 3L and 10 and 11R. Raise to 3mA. Turning on cycling-mode (90 minutes on/30 minutes off).
12 months	Switch to 50 Hz; 450 µs; 2 mA; cycling-mode (90 minutes on/30 minutes off); contact points 2 and 3L and 10 and 11R
14 months	Change to continuous stimulation at daytime and off at nighttime.
14-24 months	Continuation of 50 Hz; 450 µs; 2-3 mA

Supplementary table 2. CRS-R subscores pre- and post-DBS

	Pre-DBS				Post-DBS			
	**#1**	**#2**	**#3**	**#4**	**#1**	**#2**	**#3**	**#4**
**Auditory function scale**	2	2	1	1	1	2	2	1
**Visual functional scale**	3	3	3	3	3	3	3	3
**Motor function scale**	5	1	2	2	5	1	1	5
**Oromotor/verbal function scale**	2	2	2	1	1	1	1	1
**Communication scale**	0	0	0	0	0	0	0	0
**Arousal scale**	2	2	2	2	2	3	2	2
**Total score**	**14 (MCS-)**	**10 (MCS-)**	**10 (MCS-)**	**9 (MCS-)**	**12 (MCS-)**	**10 (MCS-)**	**9 (MCS-)**	**12 (MCS-)**

Supplementary table 3. Regions of the AAL-atlas, with cortical regions ordered according to (Gong et al., 2009).[43]

AAL atlas				
**Frontal lobe**				
1	Rectus_L		40	Rectus_R
2	Olfactory_L		41	Olfactory_R
3	Frontal_Sup_Orb_L		42	Frontal_Sup_Orb_R
4	Frontal_Med_Orb_L		43	Frontal_Med_Orb_R
5	Frontal_Mid_Orb_L		44	Frontal_Mid_Orb_R
6	Frontal_Inf_Orb_L		45	Frontal_Inf_Orb_R
7	Frontal_Sup_L		46	Frontal_Sup_R
8	Frontal_Mid_L		47	Frontal_Mid_R
9	Frontal_Inf_Oper_L		48	Frontal_Inf_Oper_R
10	Frontal_Inf_Tri_L		49	Frontal_Inf_Tri_R
11	Frontal_Sup_Medial_L		50	Frontal_Sup_Medial_R
12	Supp_Motor_Area_L		51	Supp_Motor_Area_R
13	Paracentral_Lobule_L		52	Paracentral_Lobule_R
14	Precentral_L		53	Precentral_R
15	Rolandic_Oper_L		54	Rolandic_Oper_R
**Parietal lobe**				
16	Postcentral_L		55	Postcentral_R
17	Parietal_Sup_L		56	Parietal_Sup_R
18	Parietal_Inf_L		57	Parietal_Inf_R
19	SupraMarginal_L		58	SupraMarginal_R
20	Angular_L		59	Angular_R
21	Precuneus_L		60	Precuneus_R
**Occipital lobe**				
22	Occipital_Sup_L		61	Occipital_Sup_R
23	Occipital_Mid_L		62	Occipital_Mid_R
24	Occipital_Inf_L		63	Occipital_Inf_R
25	Calcarine_L		64	Calcarine_R
26	Cuneus_L		65	Cuneus_R
27	Lingual_L		66	Lingual_R
**Temporal lobe**				
28	Fusiform_L		67	Fusiform_R

29	Heschl_L		68	Heschl_R
30	Temporal_Sup_L		69	Temporal_Sup_R
31	Temporal_Mid_L		70	Temporal_Mid_R
32	Temporal_Inf_L		71	Temporal_Inf_R
33	Temporal_Pole_Sup_L		72	Temporal_Pole_Sup_R
34	Temporal_Pole_Mid_L		73	Temporal_Pole_Mid_R
35	ParaHippocampal_L		74	ParaHippocampal_R
**Insula and cingulate gyri**				
36	Cingulum_Ant_L		75	Cingulum_Ant_R
37	Cingulum_Mid_L		76	Cingulum_Mid_R
38	Cingulum_Post_L		77	Cingulum_Post_R
39	Insula_L		78	Insula_R
**Central structures**				
79	Hippocampus_L		80	Hippocampus_R
81	Amygdala_L		82	Amygdala_R
83	Caudate_L		84	Caudate_R
85	Putamen_L		86	Putamen_R
87	Pallidum_L		88	Pallidum_R
89	Thalamus_L		90	Thalamus_R
